# A simple BLASTn-based approach generates novel insights into the regulation and biological function of type I toxin-antitoxins

**DOI:** 10.1128/msystems.01204-23

**Published:** 2024-06-10

**Authors:** Selene F. H. Shore, Michael Ptacek, Andrew D. Steen, Elizabeth M. Fozo

**Affiliations:** 1 Department of Microbiology, University of Tennessee, Knoxville, Tennessee, USA; 2 Department of Earth and Planetary Sciences, University of Tennessee, Knoxville, Tennessee, USA; Chinese Academy of Sciences, Shanghai, China

**Keywords:** toxin-antitoxin, type I toxin, *tisB*, *shoB*, *zorO*, *orzO*, sRNA

## Abstract

Bacterial chromosomal type I toxin-antitoxin systems consist of a small protein, typically under 60 amino acids, and a small RNA (sRNA) that represses toxin translation. These gene pairs have gained attention over the last decade for their contribution to antibiotic persistence and phage tolerance in bacteria. However, biological functions for many remain elusive as gene deletions often fail to produce an observable phenotype. For many pairs, it is still unknown when the toxin and/or antitoxin gene are natively expressed within the bacterium. We examined sequence conservation of three type I toxin-antitoxin systems, *tisB/istR-1, shoB/ohsC,* and *zor/orz*, in over 2,000 *Escherichia coli* strains, including pathogenic and commensal isolates. Using our custom database, we found that these gene pairs are widespread across *E. coli* and have expression potential via BLASTn. We identified an alternative, dominant sequence variant of TisB and confirmed that it is toxic upon overproduction. Additionally, analyses revealed a highly conserved sequence in the *zorO* mRNA untranslated region that is required for full toxicity. We further noted that over 30% of *E. coli* genomes contain an *orz* antitoxin gene only and confirmed its expression in a representative strain: the first confirmed report of a type I antitoxin without its cognate toxin. Our results add to our understanding of these systems, and our methodology is applicable for other type I loci to identify critical regulatory and functional features.

**IMPORTANCE:**

Chromosomal type I toxin-antitoxins are a class of genes that have gained increasing attention over the last decade for their roles in antibiotic persistence which may contribute to therapeutic failures. However, the control of many of these genes and when they function have remained elusive. We demonstrate that a simple genetic conservation-based approach utilizing free, publicly available data yields known and novel insights into the regulation and function of three chromosomal type I toxin-antitoxins in *Escherichia coli*. This study also provides a framework for how this approach could be applied to other genes of interest.

## INTRODUCTION

Tolerance to environmental stress is key for bacterial survival. The production of toxins from chromosomal toxin-antitoxin systems has been implicated in a variety of stress tolerance mechanisms, including antibiotic persistence or resistance and phage exclusion (reviewed in reference 1)]. These gene pairs encode a toxin that, when overproduced, leads to cell death, and an antitoxin which represses this toxicity. Toxin-antitoxin systems are defined by the nature (protein or RNA) of the toxin and antitoxin and the mechanism of toxin repression, ranging from type I to type VIII, with type I and type II being the most well characterized (1). For the type I toxin-antitoxin systems, these encode a small (under 60 amino acids) toxic protein which induces cellular stasis or death upon overproduction and a small RNA (sRNA) that base pairs to the toxin mRNA, preventing its toxicity (2, 3).

Initially, type I toxin-antitoxin systems were described on plasmids where they serve a role in plasmid maintenance. Later, chromosomal pairs homologous to plasmid pairs as well as chromosomal systems with no homology to plasmid sequences were identified. For many chromosomal pairs that lack plasmid homology, their true biological function is unknown. Deletion of either the toxin or antitoxin encoding gene often has no observable phenotype. This is why, following confirmation of toxicity (and repression of toxicity) via overexpression, research toward a biological function can stall. For the best described chromosomally encoded type I toxin, TisB, unraveling its function was in part possible due to identification of a conserved LexA binding site in its promoter which led investigators to perform experiments demonstrating a role for TisB during the SOS response (4). Thus, regulation knowledge can aid in functional analysis of type I toxins.

While the regulation of the TisB toxin as it relates to function is well defined, this has not been the case for many other chromosomal type I systems (5–7). One reason for this is that attempts to identify novel regulatory mechanisms bioinformatically have had limited success. This is likely due to a combination of the limits of bioinformatic identification of regulatory sequences and the fact that only a single locus is used as a DNA sequence query for such approaches. To our knowledge, no one has examined the conservation of any type I toxin-antitoxin sequence across a single bacterial species. We hypothesized that, by using such a conservation-based approach, we could identify novel features of type I systems, including regions that may contribute to regulatory function or activity.

We therefore created a custom nucleotide database of complete *Escherichia coli* genomes from National Center for Biotechnology Information (NCBI) and used a BLASTn approach to examine sequence conservation of three chromosomal type I toxin-antitoxins from *E. coli* (*tisB/istR-1*, *shoB/ohsC*, and *zor/orz*) whose functions have been either directly or indirectly implicated in antibiotic persistence or resistance (8–10). Using this approach, we confirmed that the −35 and −10 promoter elements as well as ribosome binding sites (RBS) and other regulatory regions were highly conserved for the pairs examined. We also predicted and confirmed a regulatory element within the 5′ untranslated region (UTR) of the *zor* toxin mRNA that contributes to its toxicity. Additional analyses also indicated that *tisB/istR-1* and *zor/orz* copy number correlates with intestinal disease isolates. Surprisingly, we identified that nearly a third of all *E. coli* possess an *orz* antitoxin gene without its cognate toxin gene: this provides impetus for future studies examining roles for antitoxins beyond just toxin repression.

## RESULTS

### Generation of a custom *E. coli* nucleotide database

**Fig 1:**
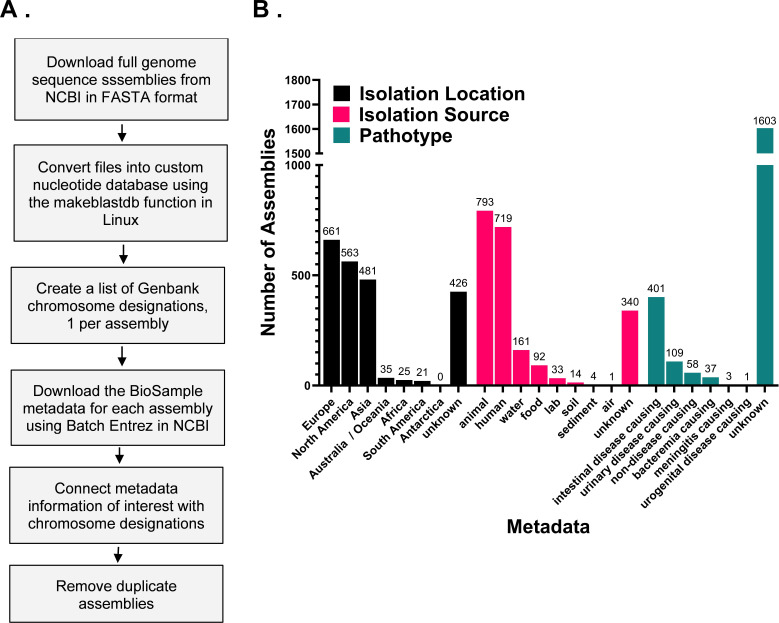
Details of custom *E. coli* nucleotide database utilized in this study. (**A**) Outline of custom database construction and (B) isolation location, sources, and pathotype of *E. coli* genome assemblies included in the final custom database.

To examine regions of high conservation as a means of identifying important regulatory sequences*,* we first created a custom database (Fig. 1A, Materials and Methods) from complete assembled genomes of *E. coli* available on NCBI (11). We designated each as an environmental or laboratory strain/isolate when possible. Environmental referred to strains isolated from known environments and included human/animal commensals or pathogenic variants. Laboratory isolates referred to strains used for cloning (e.g., K12 derivatives) or designated as laboratory/lab in their BioSample information. Following isolation source designation, we noted duplication of some specific isolates (e.g., *E. coli* MG1655) and therefore removed duplicates from all analyses, resulting in a total of 2,212 total genomes analyzed. Note that some isolates within the laboratory category were known or likely derivatives of *E. coli* MG1655 or *E. coli* B; however, we retained these derivatives for our analyses due to potential variation in the number of copies of the toxin-antitoxin systems analyzed. The breakdown of the distribution of these strains within our database, along with geographic and pathotype information (see below), can be found in Fig. 1B. Overall, the custom database used in this study represented *E. coli* with diversity in isolation location, source, and pathotype.

### MG1655 TisB is not the dominant protein variant

As proof of concept, we first examined conservation of *tisB/istR-1* as it is the best described chromosomally encoded type I toxin-antitoxin system in *E. coli*. Transcription of the toxin gene *tisB* is repressed by LexA such that expression occurs in response to DNA damage (4); the small RNA IstR-1 can base pair to the *tisB* mRNA and prevent its translation. Base pairing of IstR-1 blocks a standby ribosome binding site, which is needed for translation of *tisB* (12). If the toxin escapes mRNA repression, production of TisB results in the formation of highly tolerant cells (persister cells) to specific antibiotics (8, 13, 14). The locus also possesses *tisA*, which encodes an open reading frame (ORF) upstream of the *tisB* ORF, and encodes IstR-2, an elongated version of IstR-1 that is only transcribed during SOS response and does not repress *tisB* translation (4, 15).

**Fig 2:**
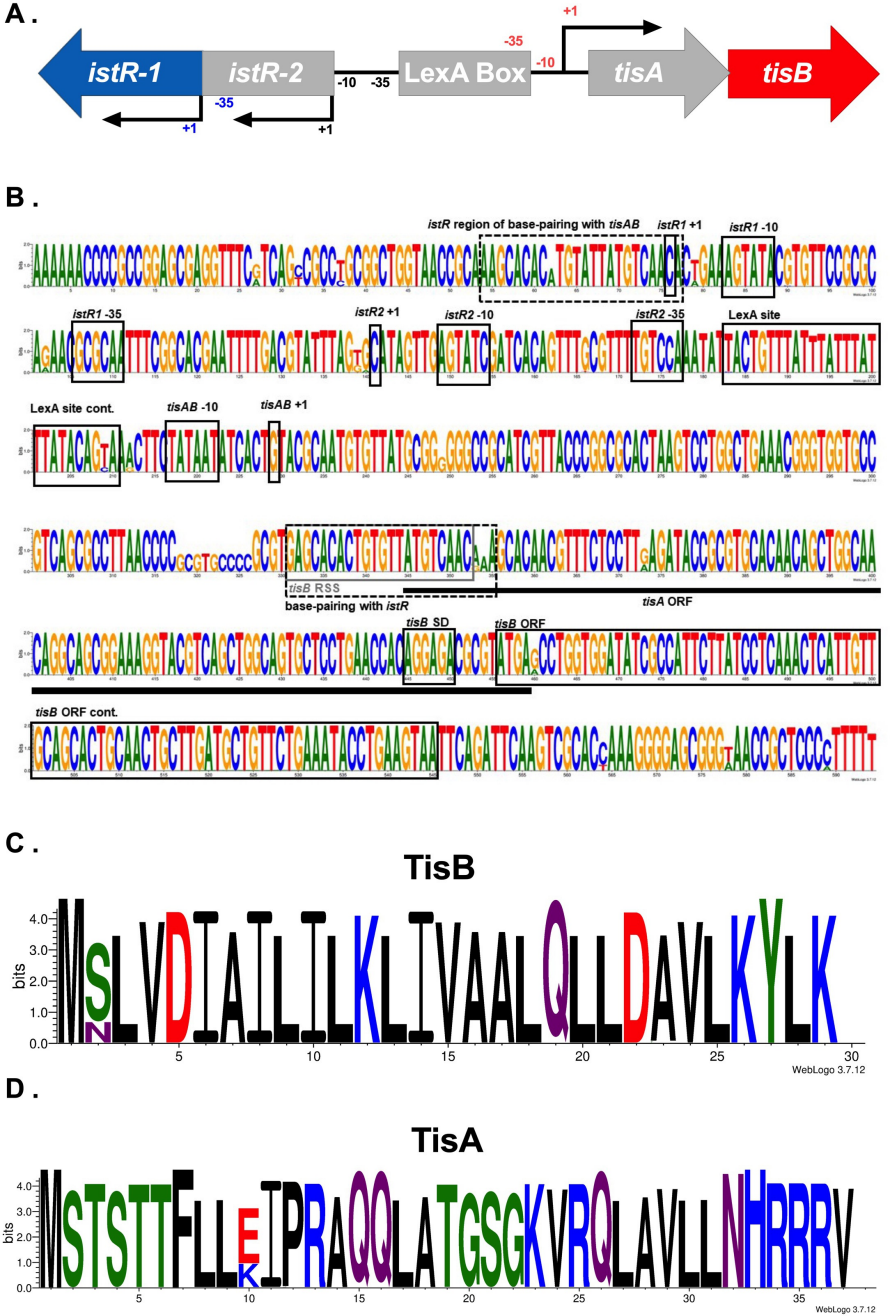
Conservation of the *tis/istR* locus across *E. coli*. (**A**) Genomic organization of *tis/istR* MG1655, (**B**) nucleotide sequence conservation of full *tis/istR* loci, (**C**) amino acid sequence conservation of TisB, and (D) amino acid sequence conservation of TisA. SD refers to the Shine-Dalgarno sequence. Note that the MG1655 amino acid sequence for TisB contains an asparagine (N) at the second position. For amino acid logos, positively charged/basic residues are in blue, negatively charged/acidic residues are in red, non-charged polar residues are in green, and hydrophobic residues are in black with neutral residues in purple.

The full locus from MG1655 containing the entire intergenic region between *tisB* and *istR-1* and all transcribed regions was used as a query for BLASTn (16), depicted in Fig. 2A [includes *tisA* and *istR-2* in the region (4); for simplicity, referred to herein as *tis/istR*]. Raw and summarized results can be found in Table 1 and Tables S1 and S2.

**TABLE 1:** Tis/IstR sequence conservation summary for full matches compared to *E. coli* MG1655

	TisB (toxin)	IstR-1 (antitoxin)	TisA (ORF)	IstR-2 (RNA)	Regulation
−35	N/A^*a*^	99.8% (TTGCGC)	N/A	94.5% (TGGACA)	Transcription
−10	99.8% (TATAAT)	100% (TATACT)	99.8% (TATAAT)	99.8% (GATACT)	Transcription
LexA (from MG1655)	93.5%				Transcription
RSS	99.9% (see Fig. 2)	N/A	N/A	N/A	Translation
RBS	100% (AGGAGA)	N/A	N/A	N/A	Translation
ORF	99.9% (29 aa)	N/A	99.6% (37 aa)	N/A	Translation

^*a*^ N/A, not applicable.

**Fig 3:**
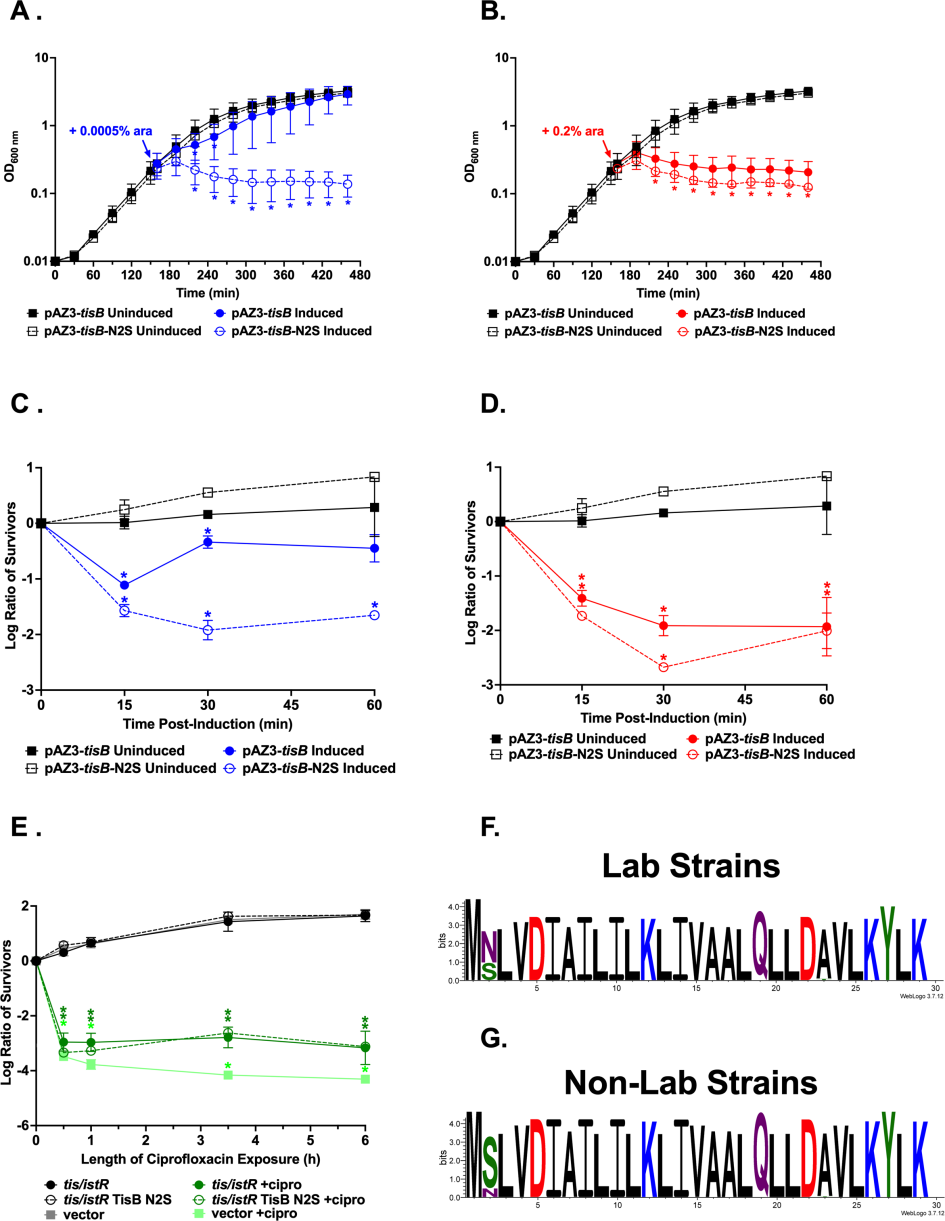
Differential toxicity between the MG1655 TisB and the consensus TisB. (**A–D**) *E. coli* UTK007 ∆*tis/istR* harboring either pAZ3-*tisB* (solid lines) or pAZ3-*tisB-*N2S (dotted lines, encoding the consensus TisB) was grown to OD_600_ ~0.25, split into two cultures as indicated by the arrow, with arabinose added to a final concentration of 0.0005% or 0.2% (induced). Note A and B represent OD_600_ values over time while C and D are the log ratios of surviving colonies over time. (**E**) *E. coli* UTK007 ∆*tis/istR* harboring either pBR322-*tis/istRmg* (solid lines) or pAZ3-*tis/istRmg-tisB-*N2S (dotted lines, encoding the consensus TisB) was grown to OD_600_ ~0.25, split into two cultures (T = 0 h), with ciprofloxacin added to a final concentration of 1 µg/mL as indicated. Shown are the averages and standard deviations for *N* = 3. * indicates a difference between induced and uninduced or treated vs untreated controls at a *P* < 0.05 calculated via multiple paired *t*-tests. (**F**) TisB consensus for lab strains and (**G**) non-lab strains.

We found that, across all *tis/istR* loci, promoter elements were highly conserved (Table 1; Fig. 2B), with 94% containing the annotated LexA binding site and −10 and −35 promoter sequences for *tisAB, istR-1,* and *istR-2* found in MG1655. The predicted ribosome standby site (RSS) and RBS for *tisB* were >99% conserved. However, the consensus TisB protein sequence (85%) contained a serine at the second position which is different from the asparagine found in MG1655 (Fig. 2C). Overproduction of this consensus TisB (*tisB*-N2S) from an arabinose-inducible promoter on a multicopy plasmid revealed that *tisB*-N2S was more toxic than the MG155 *tisB* when induced at lower arabinose levels (0.0005%). Induction at high levels of arabinose (0.2%) resulted in equivalent toxicity between TisB and TisB N2S (Fig. 3A through D). There was no difference in cell survival over a 6-h exposure to ciprofloxacin when *tis/istR* from MG1655 with TisB or TisB N2S was present on a multicopy plasmid (Fig. 3E), suggesting the increased toxicity did not impact persister cell formation.

We further examined whether TisB from MG1655 is a lab strain-specific protein variant by examining conservation of TisB in lab vs non-lab strains. Lab strains from the K-12 lineage contain TisB while those from the B lineage contain TisB N2S (Fig. 3F through G; Table S2). These data suggest that when the lab strains were originally isolated from their environmental host (see references 17–19 for lab strain ancestry), they contained one of the naturally occurring TisB variants.

Despite no experimental evidence that the *tisA* ORF is translated, >99% of *E. coli* containing a *tis/istR* locus encoded the 37 amino acid ORF with high protein sequence conservation (Fig. 2D). The biological implications of this are currently unknown.

### High conservation of predicted regulatory elements of the *shoB/ohsC* locus across *E. coli*

**Fig 4:**
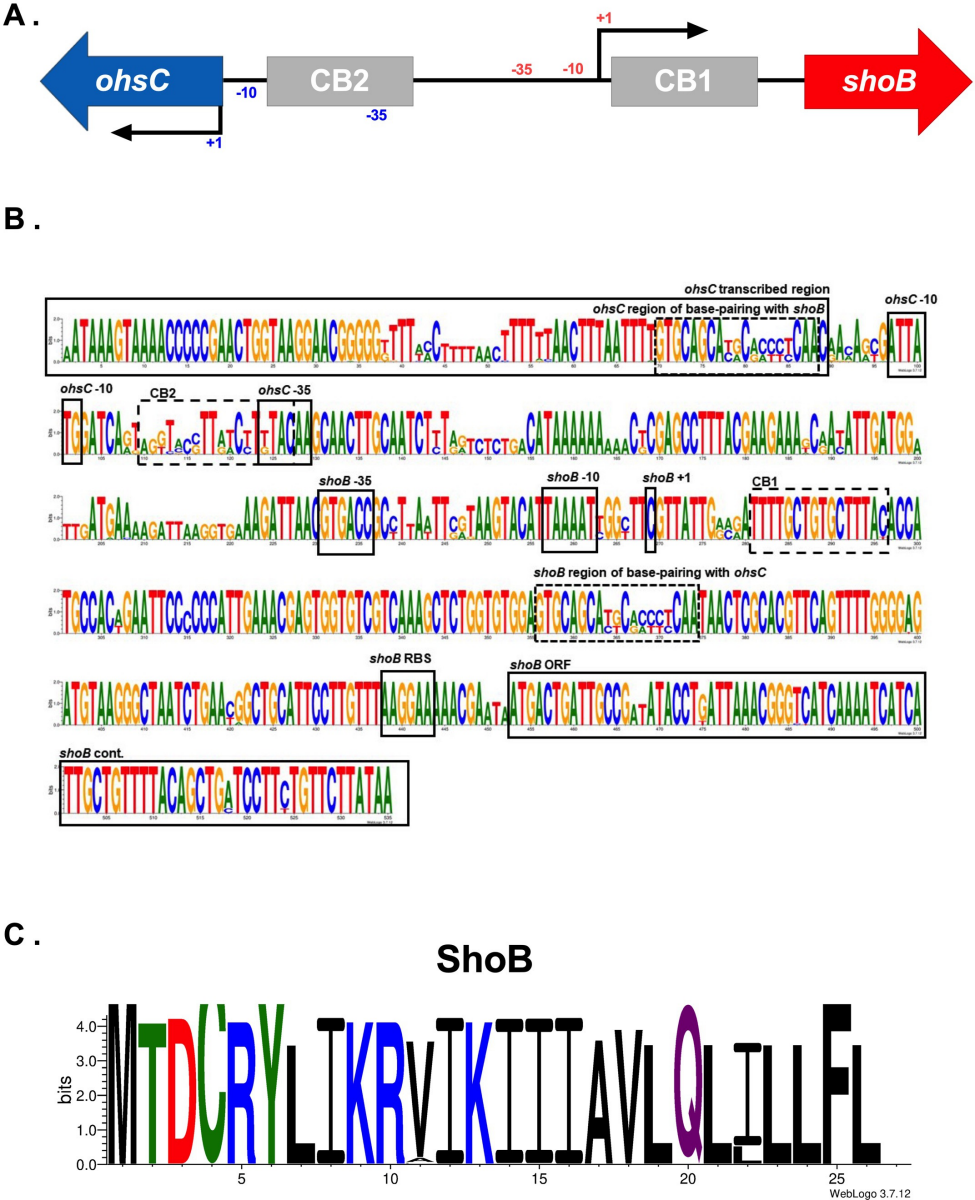
Conservation of the *shoB/ohsC* locus across *E. coli*. (**A**) Genomic organization of *shoB/ohsC* MG1655, (**B**) nucleotide sequence conservation of full *shoB/ohsC* loci and (C) amino acid sequence conservation of ShoB. For amino acid logos, positively charged/basic residues are in blue, negatively charged/acidic residues are in red, non-charged polar residues are in green, and hydrophobic residues are in black with neutral residues in purple.

Given the strong conservation observed for the LexA binding site above, we examined whether known transcription factor binding sites were conserved in another type I toxin-antitoxin locus. The chromosomal type I toxin-antitoxin *shoB/ohsC* (20, 21) has been indirectly implicated in the ability of *E. coli* to survive the antibiotic colistin (10). Like *tis/istR*, this locus contains a type I toxin gene, *shoB*, and an antitoxin gene, *ohsC*. The transcription factor CpxR was found to regulate *shoB* and *ohsC* at two proposed binding sites, named Cpx binding (CB1) and CB2 (22). However, it is not known whether binding of CpxR to one or both CB sites regulates *shoB* and/or *ohsC in vivo*. We used the *shoB/ohsC* sequence from MG1655 as our query (Fig. 4A), to examine the presence of CB site (20, 23), the results of which can be found in Table 2 ; Tables S3 and S4.

**TABLE 2:** ShoB/OhsC sequence conservation summary compared to *E. coli* MG1655

	ShoB (toxin)	OhsC (antitoxin)	Regulation
−35	99.9% (GTGACC)	66.8% (TTGTAA), 33.1% (TTGTAC)	Transcription
−10	99.6% (TAAAAT)	100% (CATAAT)	Transcription
CB1 (from MG1655)	91.3%		Transcription
CB2 (from MG1655)	52.2%		Transcription
RBS (AAGGAA)	100%	N/A	Translation
ORF (26 aa)	>99.9%	N/A	Translation

Conservation of CB1 across strains was high with 91% having the identical sequence of MG1655 (Table 2; Fig. 4B). In contrast, only 52% of loci had the same CB2 as MG1655 with the region encompassing CB2 being highly variable. Additionally, except for the −35 of *ohsC* which overlaps with CB2, >99% of *shoB/ohsC* had the identical promoter elements, RBS, and ORF length for *shoB* and *ohsC* as MG1655. The consensus ShoB amino acid sequence can be found in Fig. 4C.

### High conservation of type I toxin-antitoxin *zor/orz* reveals a novel sequence that controls ZorO-induced toxicity

**Fig 5:**
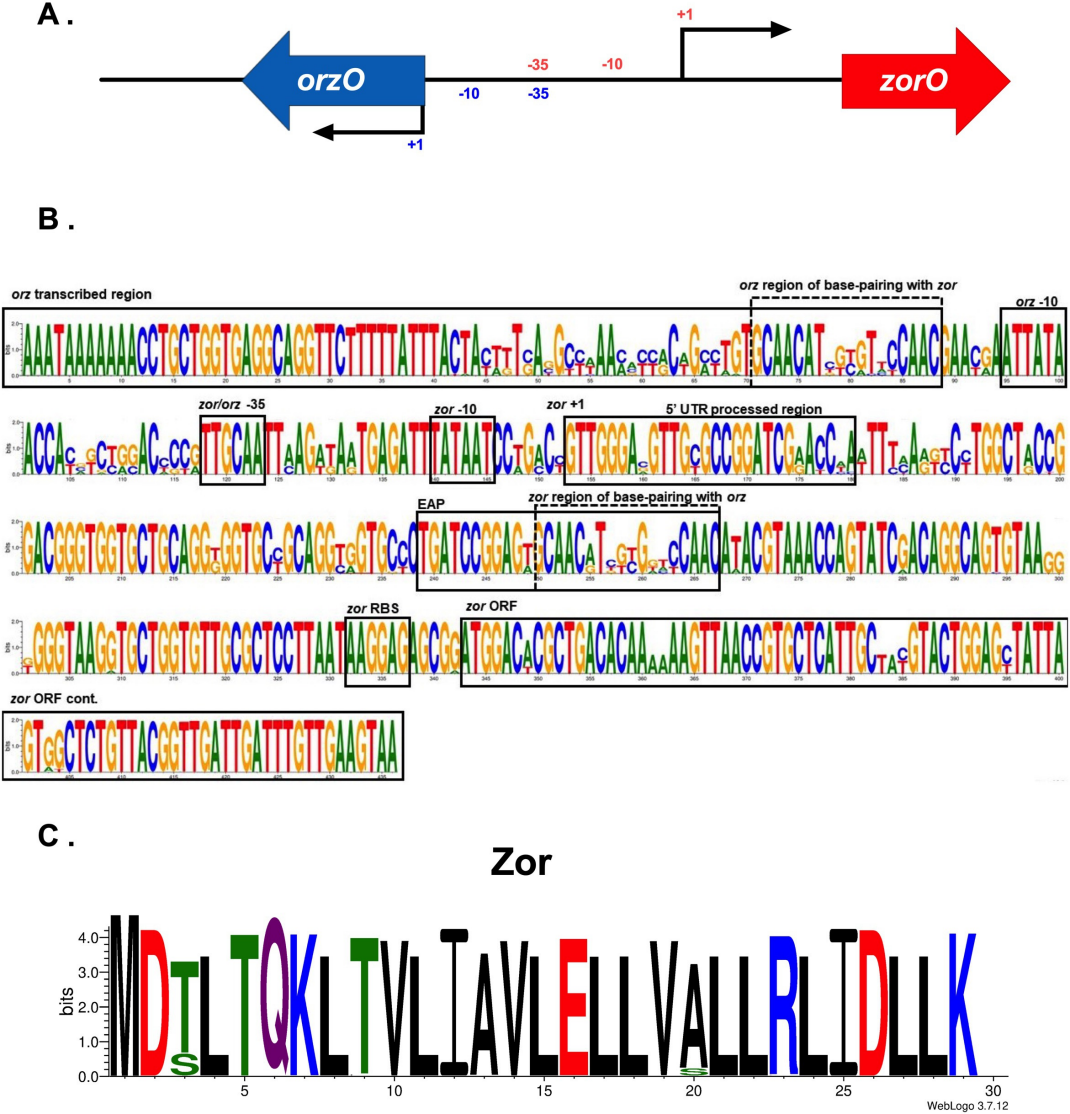
Conservation of the *zor/orz* locus across *E. coli*. (**A**) Genomic organization of *zor/orz* EDL933, (**B**) nucleotide sequence conservation of full *zor/orz* loci (includes all copies in strains containing more than one *zor/orz* copy), and (C) amino acid sequence conservation of Zor. For amino acid logos, positively charged/basic residues are in blue, negatively charged/acidic residues are in red, non-charged polar residues are in green, and hydrophobic residues are in black with neutral residues in purple.

Finally, we wanted to examine whether this approach could be used in a system not identified in *E. coli* MG1655 but possessed in a pathogenic variant, *E. coli* O157:H7 EDL933 (referred to herein as EDL933). The *zor/orz* locus was initially identified in 2010 as two tandemly encoded, highly similar toxin-antitoxin pairs, *zorO/orzO* and *zorP/orzP*, with a *zor* gene encoding a 29 amino acid toxin protein and an *orz* gene encoding the antitoxin (21, 24). Translation of *zorO* is repressed by its own 5′ UTR (25). Processing of the 5′ UTR results in a single-stranded open region (termed EAP, for “exposed after processing,”) far upstream of the ribosome binding site. This EAP region is required for robust translation but is also where the OrzO antitoxin binds. Multiple copies of the *zor-orz* locus from EDL933 or just the *zorO-orzO* pair increased the minimum inhibitory concentration of aminoglycoside antibiotics and reduced lag in sublethal levels of kanamycin, though the mechanism is unknown (9, 26). We initially examined *zor/orz* sequence conservation with *zorO/orzO* from EDL933 as a query (depicted in Fig. 5A). Follow-up queries are described in Materials and Methods.

Across all *zor/orz*, promoter elements were highly conserved with 99% or more loci containing the same −10 and −35 promoter sequences as found in EDL933 (Table 3; Tables S5 to S10; Fig. 5B). ZorO, the product of the *zorO* gene from EDL933 (21), was the dominant protein sequence identified from 72.5% of all *zor* ORFs. Major protein variants identified also included ZorP and ZorQ with amino acid differences relative to ZorO of T3S and A20S, respectively (Fig. 5C). In this case, ZorP refers to the product of the *zorP* gene from EDL933 (21), while ZorQ represents a common protein variant of a third locus type, *zorQ/orzQ* (see Materials and Methods).

**TABLE 3:** Zor/Orz sequence conservation summary for full matches to *zor/orz* or *orz*-only loci compared to EDL933^*a*^

	Zor (toxin)	Orz (antitoxin)	Regulation
Full *zor/orz* loci			
−35 (TTGCAA)	99.4%	99.4%	Transcription
−10 (TATAAT)	100%	99.8%	Transcription
Base-pairing potential (based on Wen et al. [24])	99.8%		Post-transcription
RBS (AAGGAG)	99.8%	n/a	Translation
ORF (29 aa)	99.5%	n/a	Translation
*orz*-only loci			
−35 (TTGCAA)	99.7%	99.7%	Transcription
−10 (TATAAT)	100%	98.9%	Transcription

^*a*^ A total of 2,439 full *zor/orz* were analyzed and 656 *orz*-only loci were analyzed.

The region of base pairing between the *zor* mRNA and Orz sRNA was quite variable. This variation may prevent crosstalk between *zor/orz* loci in multicopy strains as is the case for *zor-orz* from EDL933 (24). We previously demonstrated that either 15 nucleotides of continuous base pairing or 17 nucleotides of discontinuous base pairing with one internal mismatch was sufficient for OrzO to repress ZorO-induced toxicity (24). Using these as requirements for repressive activity, we determined that 99.8% of Orz in full *zor/orz* have the potential to repress their cognate *zor* (Table S11).

**Fig 6:**
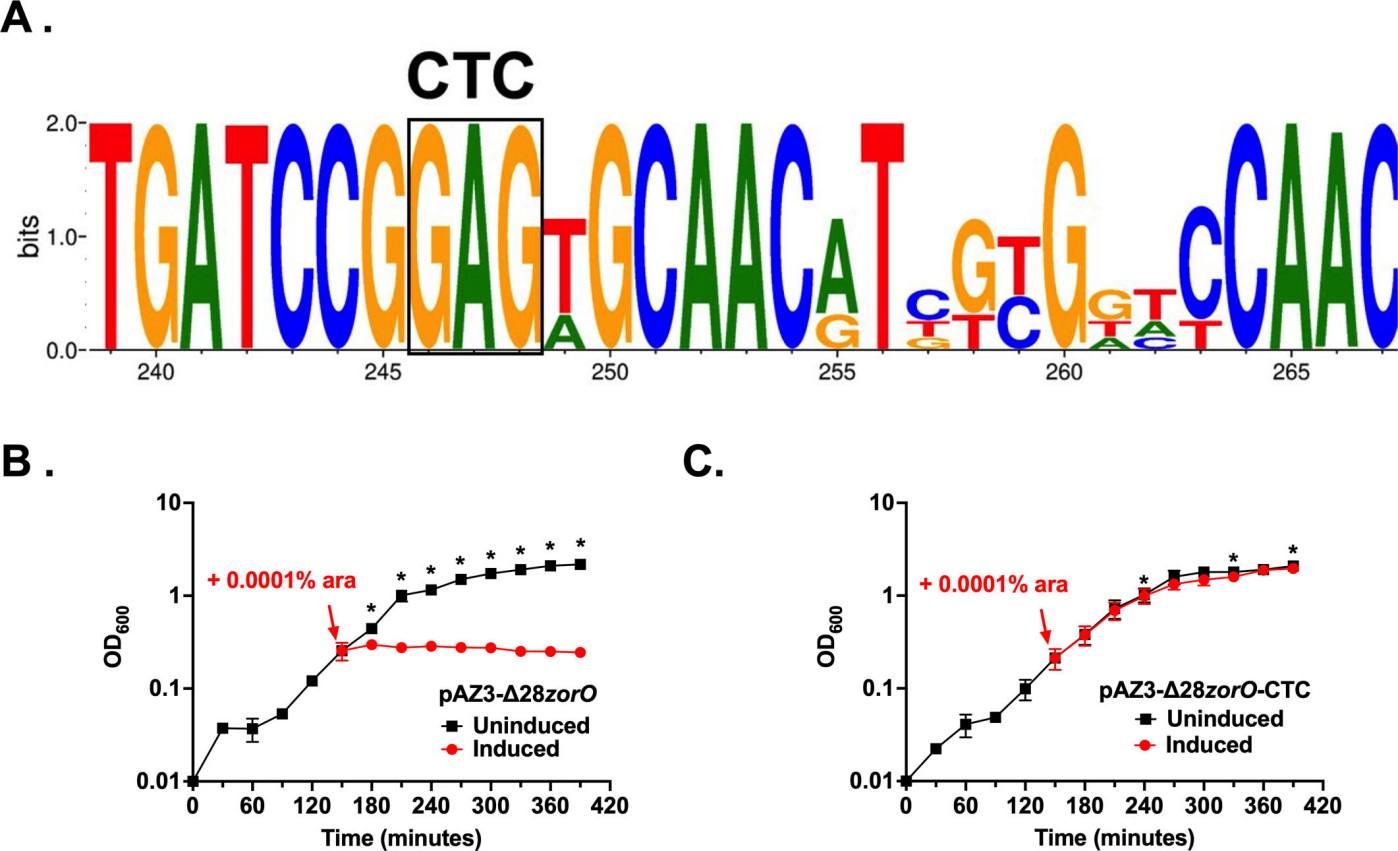
Identification of critical residues for toxicity in the 5´ UTR of *zor*. (**A**) DNA consensus from all *zor/orz* for the EAP region originally identified in *zorO* from strain EDL933. Nucleotides 245–250 represent a possible ribosome standby site. GAG (boxed) indicates sequence mutated to CTC in pAZ3-Δ28*zorO*-CTC. (**B**) *E. coli* UTK007 harboring either pAZ3-Δ28*zorO* or pAZ3-Δ28*zorO*-CTC was grown to OD_600_ 0.25, split into two cultures as indicated by the arrow, and arabinose was added to a final concentration of 0.0001% (induced). Shown are the averages and standard deviations for *n* = 3. * indicates a difference between induced and uninduced controls at a *P* < 0.05 calculated via multiple paired *t*-tests.

The EAP region of *zorO* was hypothesized to contain a ribosome standby site similar to the *tisB* mRNA that allows for increased ribosomal interaction and translation of the toxin (2, 12, 27, 28). While ribosomal binding to the EAP of *zorO* has not been demonstrated, removal of a portion of the EAP sequence did reduce translation efficiency (25). We noted that, within the *zor/orz* EAP consensus, there was a conserved “GGAGTG/AG” sequence that resembled a ribosome binding site (Fig. 6A). We mutated the GAG of this sequence to CTC in a plasmid that overproduces the toxic-processed (Δ*28zorO*) variant from EDL933 and compared its overproduction to the wild-type sequence. As shown in Fig. 6B and C, mutation of these residues reduced toxicity compared to the parental plasmid.

### Copy number analysis reveals presence of widespread *orz* antitoxin-only strains

It was previously suggested that copy number of toxin-antitoxins may correlate with pathotype, but given a limited number of complete *E. coli* genomes at the time, a thorough investigation was not performed (21). To test the hypothesis that copy number varies by pathotype, we first quantified the variability in copy number across all *E. coli* in our database.

Eighty-two percent of *E. coli* had one full match to *tis/istR*, 3% had an interrupted match, and 15% had no match to *tis/istR*. For *shoB/ohsC*, *E. coli* contained either a full (98%), interrupted (1%), or no match (<1%) to *shoB/ohsC*. In addition, two antitoxin-only strains were identified for *shoB/ohsC*. Copy number designations and evidence of insertion or deletion events for interrupted and antitoxin-only strains were noted in Tables S2, S4, and S10.

**Fig 7:**
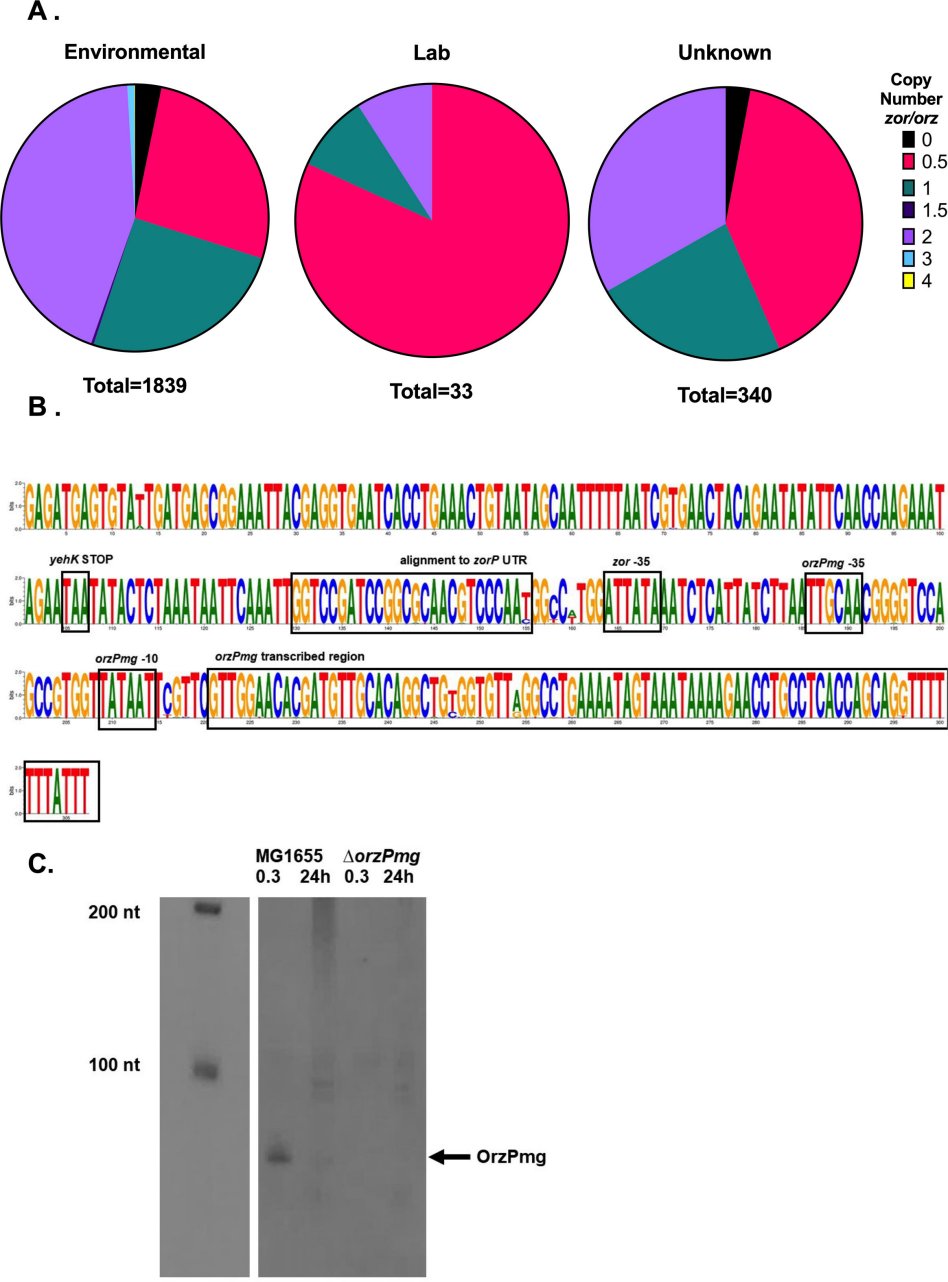
Evidence of antitoxin-only strains. (**A**) Copy number variation for *zor/orz* in lab and environmental strains of *E. coli* detected via BLASTn. Copy number of 0.5 refers to containing one *orz*-only locus; a copy number of 1 has one *zor/orz* locus; a copy number of 1.5 has one *zor/orz* locus and an additional *orz*-only locus, etc. (**B**) Nucleotide sequence conservation of *orz* loci in *orzP*-only strains. (**C**) OrzPmg detection in MG1655. Northern blot of total RNA from strain MG1655 or the Δ*orzPmg* derivative isolated from cells grown to OD_600_ 0.3 and 24 h post-inoculation. Shown is a representative of three biological replicates.

For *zor/orz,* we found that 3% of the 2,212 assemblies contained no match to *zor/orz*, 25% had one full match to *zor/orz* (i.e., a single toxin-antitoxin pair), 42% had two full matches to *zor/orz,* and <0.1% had a disrupted locus (Fig. 7A; Table S10). A minority of genomes were unusual in their copy number for *zor/orz*. Sixteen had three full *zor/orz* loci, six had one full locus with an additional *orz* (1.5 copies), and an isolate from a human patient contained four copies of *zor/orz* on its chromosome. Unlike for *tis/istR1* and *shoB/ohsC*, we did identify a two-copy *zor/orz* locus on a plasmid (p11A_p2) from a hospitalized patient, though these were identical to the chromosomal copies (Table S10).

Surprisingly, approximately 30% of genomes lacked an intact *zor* toxin gene but possessed solely an antitoxin (*orz*) gene. In >99% of these *orz*-only assemblies, 27–29 nucleotides of the *zor* 5′ UTR was still present as determined via an alignment to *zorP* from EDL933. Since these fragments of the *zor* UTR did not contain the toxin open reading frame nor did they possess the base-pairing region for Orz interaction, these strains were considered to be “*orz-*only” (antitoxin only; 0.5 copies). In 96% of cases, these *orz*-only loci share highest similarity to *orzP* from strain EDL933 and were highly similar to each other (Fig. 7B).

Given this finding, we wanted to determine if an orphan *orzP* was expressed. We examined dRNA-Seq of MG1655 published previously and noted a possible transcript in this region of MG1655 (an *orz-*only variant [29, 30]). We confirmed this via northern analysis in MG1655 and a derivative deleted for the gene (Fig. 7C). Given that the sequence in MG1655 was 100% identical to the previously identified OrzP antitoxin from EDL933, we named this sRNA OrzPmg.

We also detected 18 partial matches on plasmids to a ~113 nucleotide segment of the *zor* 5′ UTR, but this segment, which ranged from 71% to 74% identity to the analogous *zor* sequence, was ~35 nucleotides upstream of a *dinQ* toxin ORF, suggesting that this was in fact part of a potential *dinQ* 5′ UTR and not a *zor* 5′ UTR (Table S6). To our knowledge, no evolutionary relationship has been proposed between the *zor/orz* and the *dinQ/agrB* toxin-antitoxin families.

### Metadata analysis of *zor/orz* and *tis/istR* provides potential link between copy number and pathotype

**Fig 8:**
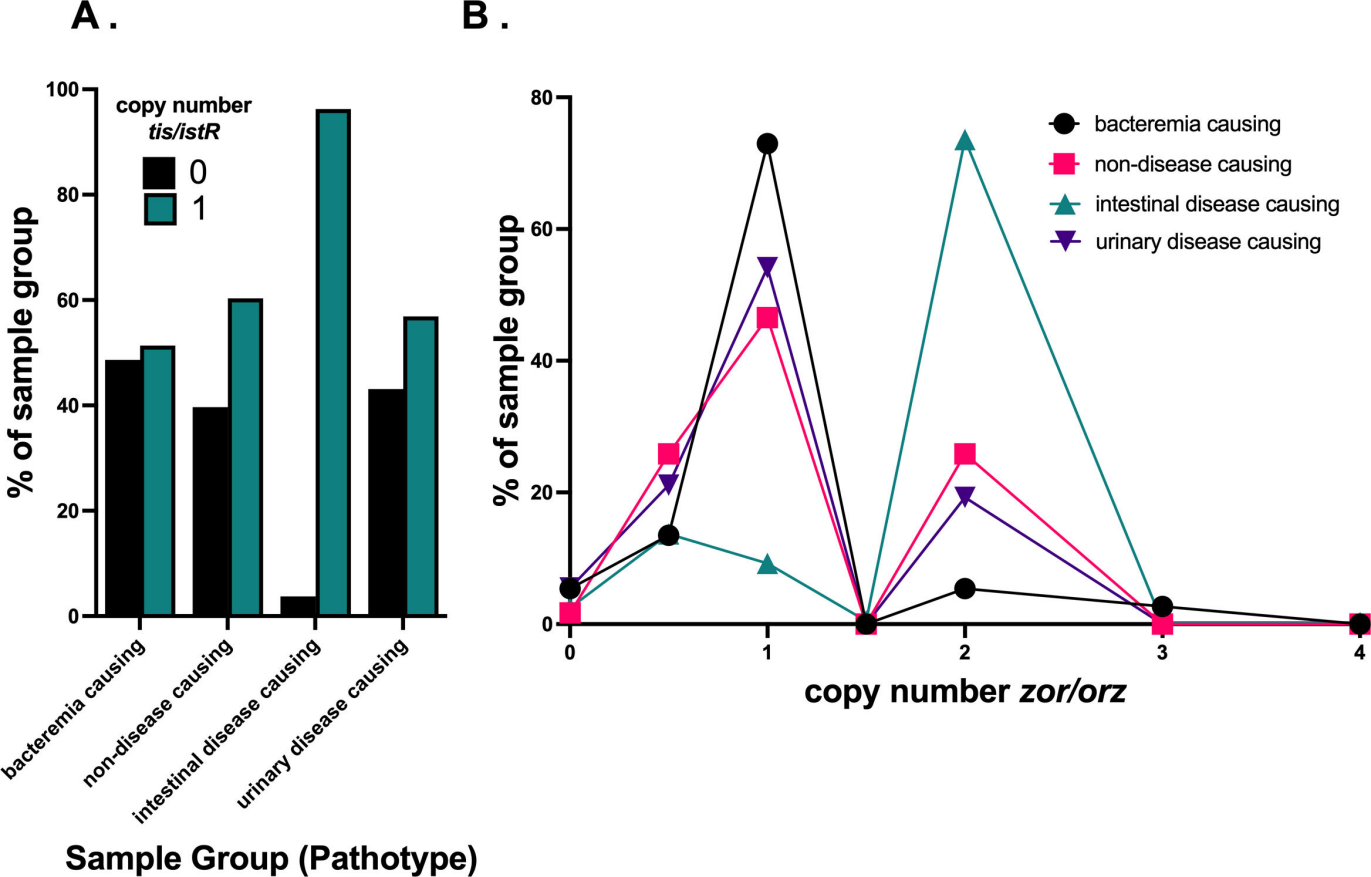
Copy number variation in environmental *E. coli* strains by pathotype for (**A**) *tis/istR* and (**B**) *zor/orz*. For pathotype designation, the category refers to the disease the sample can cause in humans (see Materials and Methods). Healthy samples came from human stool, rectal, or other gastrointestinal sources that were designated as being from a healthy host.

Given that we detected notable variation in copy number for *tis/istR* and *zor/orz* but minimal variation for *shoB/ohsC*, we sought to determine whether copy number would vary by general pathotype for *tis/istR* and *zor/orz*. We indeed found that pathotype correlated with differences in copy number variation for *tis/istR* and *zor/orz* (Fig. 8). *Post hoc* analysis using a Monte Carlo Tukey test indicated that differences in copy number among pathotypes were driven by the intestinal disease-causing isolate group: intestinal disease-causing isolates were both more likely to have *tis/istR* (*P* < 0.05 for all comparisons; *n* = 605, 10^4^ resampling iterations) and to have more copies of *zor/orz* (*P* < 0.05 for all comparisons, *n* = 605, 10^4^ resampling iterations) than the other pathotype groups analyzed. We found that there may be a correlation in *zor-orz* copy number distribution dependent upon geographical isolation, but these differences were modest (Materials and Methods).

### BLASTn identifies *zor/orz* genes that PSI-BLAST and tBLASTn do not

We also compared the BLASTn-based method employed in this study to the most commonly used bioinformatic approaches to identify type I toxin-antitoxin loci, i.e., PSI-BLAST and tBLASTn, which use only toxin amino acid sequences as the query. We found that, while tBLASTn was able to detect most Zor ORFs (seven were undetected; see Materials and Methods for settings), PSI-BLAST failed to detect the majority of Zor ORFs (Tables S12 to S15). This is likely because PSI-BLAST relies on prior annotation of ORFs which, when performed automatically, often miss small ORFs. Neither could detect *orz*-only loci as these loci did not contain an ORF. It is important to note that PSI-BLAST and tBLASTn did not detect *zor* toxin genes that were not also detected via BLASTn. Together, a BLASTn may be a more sensitive approach to identifying both type I toxin and antitoxin genes in a single species of interest.

## DISCUSSION

The initial goal of our study was to investigate whether nucleotide conservation of type I toxin-antitoxins could identify novel sequences related to the regulation and possible function of these loci. By using a custom genomic database for over 2,000 *E. coli* strains, we identified conserved sequences and features for three chromosomally encoded type I toxin-antitoxin systems. We were able to correlate copy number of *tis/istR* and *zor/orz* to intestinal disease-causing isolates, as well as uncovered the widespread presence of an antitoxin-only locus (*orz*-only) that is conserved across environmental and laboratory strains of *E. coli*. We validated expression of this antitoxin via northern analysis in an *orz*-only strain.

The results of this study highlight the power of BLASTn-based conservation approaches for identifying known type I toxin-antitoxin loci within a single species of interest. For example, BLASTn detected genes from these loci that had not been detected previously, such as for *orz* antitoxins in *orz*-only strains that were not detected via PSI-BLAST or tBLASTn. This nucleotide-based conservation approach generated hypotheses for previously described toxin-antitoxin pairs. While it was proposed that the *zor* 5′ UTR contained a ribosome standby site (25), it was not testable until the consensus generated in this approach revealed a sequence reminiscent of an RBS. This observation allowed for targeted disruption of that sequence to identify its impact on toxicity (Fig. 6C). Therefore, the use of simple bioinformatics approaches can provide needed insight into regulatory features of a type I toxin-antitoxin pair.

We were surprised to note that, often, >99% of −35 and −10 promoter elements for these toxin-antitoxin genes across the strains in our database were identical to the reference query genome (MG1655 or EDL933). To our knowledge, no one has examined sequence conservation of these specific elements for a gene across a single species. We used our BLASTn approach for three other non-essential σ^70^-controlled genes (*sodA, araC*, *ompC*) in MG1655 and compared their −10 and −35 sequences across the isolates in our database (see Materials and Methods). We found that for the matches obtained, 100% had the exact same −10 and −35 promoter elements (Tables S16 to S19). Thus, the high levels of conservation for the type I toxin-antitoxins examined was not outside the norm for σ^70^-controlled, non-essential genes. It should be noted that, even though these three genes were considered to be non-essential for *E. coli* (31), 99.1%–99.8% of the strains within our database contained at least one full match to each after identification via BLASTn. Therefore, it is possible that these genes conferred a fitness advantage in nature which could impact conservation estimates.

While several questions relating to the regulation of these three toxin-antitoxin systems in *E. coli* were addressed by the results of this study, what perhaps was more impactful was the new questions this study generated. One is whether a type I antitoxin could be performing a function in the absence of a type I toxin. A significant portion of genomes analyzed contained only an *orz* antitoxin sequence fully intact. We noted as well that a large portion of lab strains are *orz* only. When examining the history of *E. coli* lab strain variants, it appears that most are derived from a few (possibly only one or two) original isolates (17–19). Thus, this enrichment is likely due to chance as some non-lab *E. coli* strains possess only *orz*.

In *orz*-only strains, both the transcriptional regulatory sequences and the transcribed region itself are highly conserved. In *E. coli* MG1655, the promoter sequence and transcribed region for *orzPmg* are 100% identical to the *orzP* gene found in pathogenic *E. coli*. We confirmed the presence of this RNA both via direct northern analysis and noted its detection by others via dRNA-Seq (29, 30). No evidence in the literature suggests type I antitoxins may function beyond toxin repression. However, NikS in *Helicobacter pylori* is a small RNA that regulates major virulence factors; it is also proposed to be a type I antitoxin to *aapB* (32, 33). Combined with OrzPmg, it suggests that at least some antitoxins have functions beyond toxin repression. We note that several other antitoxins are readily detected under laboratory conditions, even when their toxin mRNAs are not easily detectable (5, 23).

It is not surprising that we see evidence of antitoxin-only strains but not toxin-only strains. In the absence of a toxin gene, antitoxin presence would theoretically cause no negative effects on the cell. However, if there is only a toxin gene without its cognate antitoxin, this deregulation of the toxin could result in increased levels of toxin production, resulting in potential growth inhibition or cellular death. This has even been experimentally validated for some chromosomal type I toxin-antitoxins. For example, under constitutive SOS-inducing conditions, deletion of the chromosomally encoded *istR* antitoxin was nonviable unless *istR* was also present on a plasmid (4). For the *ratA/txpA* type I toxin-antitoxin in *Bacillus subtilis*, deletion of the *ratA* antitoxin resulted in a lysis phenotype during late stages of colony growth while deletion of *txpA* with *ratA* gave no lysis phenotype (34).

Using this approach, we noted widespread conservation for the three toxin-antitoxin systems across *E. coli* lab and environmental strains. Past analyses of *hok/sok* copies found in *E. coli* chromosomes indicated variation in their potential for expression given various insertional elements within the copies and differences in regulatory features (35–37). The high degree of regulatory feature (and protein) conservation for *tis/istR*, *shoB/ohsC*, and *zor/orz* suggests that these systems may possess a true biological function. Furthermore, we noted that, for the minimal lab strains present in our studies, there does not appear to be “loss” of gene expression potential, at least in the time since laboratory domestication. However, more systematic biological analyses of environmental and lab species are needed.

We do appreciate the limitations of our analysis. As our analyses are focused upon sequence conservation, we may be missing new insights into toxin-antitoxin biology given regions of high-degree sequence variation. For example, there is large variation in the regions of base pairing for *shoB-ohsC* (Fig. 4B) and for *zor-orz* (Fig. 5B). As there was experimental evidence for the requirements of *zor-orz* base pairing (24), we were able to validate that for an individual *zor-orz*, the majority possess enough pairing potential for successful regulation. The same cannot yet be said for *shoB-ohsC*. Another important limitation is that the sequences deposited within NCBI may be biased in favor of strains that cause disease and some projects may have sequenced multiple *E. coli* isolates from related populations. Additionally, since not all strain designations were included in the BioSample metadata information, it is possible that not all duplicate sequences were removed. Therefore, one must be careful about trying to apply conservation estimates obtained this way to the entire population of *E. coli*. However, our BLASTn approach provides a framework for capturing major trends.

We also appreciate that other approaches could be applied to further probe conservation of these loci in our database. For example, the *orz* antitoxins (originally sRNA-1 and sRNA-2) were identified using RNA secondary structure prediction (21). For *tis/istR* and *zor/orz*, RNA structural analyses have been performed; however, we note that structure prediction software did not accurately predict the structure for the processed form of *zor* (25). While all three toxin mRNAs are processed in order to be effectively translated, it is still unknown what does the processing: an RNase, multiple RNases, or self-cleaving RNAs are all potential possibilities. Given these unknowns and that we wanted to create an approach that can be done with publicly accessible data by those with limited available tool sets, a nucleotide BLAST approach can still be informative.

Overall, we have demonstrated the benefits of utilizing a custom nucleotide database to detect type I toxin-antitoxin loci which are notoriously difficult to identify given the limitations of protein-based algorithms. From our approach, we identified a large portion of *E. coli* strains harboring the type I antitoxin gene *orz* without its cognate toxin gene, an observation that was missed in previous studies and represents the first known widespread finding of a solo type I antitoxin. This approach also allowed us to identify critical regulatory features and develop new hypotheses for these loci. Moving forward, combining multiple conservation approaches will likely expedite identification of loci and important features across bacteria.

## MATERIALS AND METHODS

### Custom *E. coli* nucleotide database construction and BioSample metadata

*E. coli* sequence assemblies were obtained by searching for “*E. coli*” in the NCBI Assembly database using filters for non-anomalous, non-partial, complete genomes on 31 August 2021 (11). These assemblies were then compiled into a custom database of complete *E. coli* using the makeblastdb utility of the NCBI BLAST toolkit (version 2.9.0+) run on Linux (Ubuntu 20.04.6 LTS). After further analysis, we removed one assembly (GCA_902141745) from our custom database because of its small size (170,000 bp), even though it was labeled as a complete genome.

Some common lab strains such as strain MG1655 were represented multiple times in the custom BLASTn database. Therefore, all assemblies with the same strain name were compared for *tis/istR, shoB/ohsC*, and *zor/orz* copy number. If all strains with the same name also had the same copy number for these toxin-antitoxins, one was labeled as “duplicate, keep” and maintained for analyses, while all others were labeled as “duplicate, remove” and not included in analyses (Table S20). In a single case, two assemblies with the same strain name had a difference in copy number. For the three DH5α strains, two assemblies had 0.5 copies of *zor/orz* (i.e., they harbor only *orz*) while one assembly had two copies, so one genome with 0.5 copies was retained and one genome with two copies was retained for analyses. See Table S1 notes section for duplicates removed. This resulted in a custom nucleotide database of 2,212 *E. coli* genome assemblies.

### Metadata collection and analysis

Metadata information for each assembly in the custom database was collected by downloading all corresponding BioSample data from NCBI using utilizing GenBank accessions as queries for the Batch Entrez tool (11). BioSample data were organized and linked to the custom database nomenclature manually in each table. Each strain was then categorized as either environmental or laboratory based on isolation source and pathotype information and connected to its chromosomal GenBank code. We called strains “environmental” if isolated from humans, animals, water, food, plants, terrestrial, sediment, and air; note that this category included pathogens and commensals. “Laboratory” isolates included those with strain names of known laboratory strains (such as K12 derivatives) and those with “laboratory” in their metadata for isolation source. A full list of assemblies categorized as “lab isolates” can be found in Table S20. Assemblies with unknown isolation sources were categorized as unknown.

### BLASTn query sequences and parameters

BLASTn methods were utilized throughout this study (16). All query sequences are included in Table S21. For *tis/istR*, the transcribed region of *tisB* from MG1655, with the shared intergenic region with *istR* through the *istR* transcribed region, was used as a query (4) (Fig. 2). For the *shoB/ohsC* locus, the sequence including the *shoB* ORF, the intergenic region between *shoB* and *ohsC*, and the *ohsC* transcribed region was used as a query (20) (Fig. 4). To perform BLASTn searches described for *zor/orz,* the “blastn” program from the NCBI BLAST toolkit was run using text files of the *zorO/orzO*, UTR-*zorO*, and *orzO* DNA sequences from EDL933 (24). Query “*zorO/orzO*” refers to the full *zorO/orzO* locus containing the predicted transcribed region of *orzO* through its shared intergenic region of *zorO* to the *zorO* stop codon (Fig. 5). The second query “UTR-*zorO* only” was from the mapped *zorO* transcriptional start site to its stop codon (9, 21). The third query, “*orzO* only,” contained the predicted transcribed region of *orzO* (21). After analysis of copy number was performed, the full *zorP/orzP* locus containing the predicted transcribed region of *orzP* through its shared intergenic region with *zorP* and to the *zorP* stop codon was used as query to confirm locus copy number. For BLASTn, queries were provided in FASTA format and the BLASTn setting was set to 10,000 max sequences (-max_target_seqs), much larger than the possible number of loci in each database, with task set to “blastn.” Additional default settings were used, including an E-value cutoff of 10.

### Analysis of *zor/orz, tis/istR*, and *shoB/ohsC* loci for conservation and evidence of gene expression potential

The protein sequence for the toxin ORF was determined by translating the BLASTn results for each match using the Sequence Manipulation Suite Translate tool (38). Predicted −10 and −35 promoter boxes, RBS, RSS, and relevant transcription factor binding sites (LexA, CpxR [4, 22]) were determined using manual curation of BLASTn results with the assistance of Jalview as a user interface (39). The identification of these sites was based on previous annotation (4, 20, 22–25, 28) and were categorized as either the same as in MG1655 (for *tis/istR* and *shoB/ohsC*) or EDL933 (for *zor/orz*). Consensus sequence logos were constructed using WebLogo 3 (40). Base-pairing potential was determined manually with the assistance of Jalview as a user interface (39).

### Analysis of BLASTn results for toxin-antitoxin copy number

*tis/istR* and *shoB/ohsC* copy number was determined via manual curation of the BLASTn results. BLASTn picked up additional short matches to the 3′ end of *istR* (~25 nucleotides in length) and *ohsC* (~51 nucleotides in length). When looking at a few of these examples in-depth, it was discovered that the short *istR* matches do not appear near any predicted small ORF and may represent a somewhat common sequence for transcriptional termination in *E. coli*. Follow-up analysis of the surrounding genomic region found no evidence that the short *istR* matches represented interrupted *tis/istR* loci or *istR*-only loci and were therefore not considered for conservation or copy number analysis. The short *ohsC* matches appeared to represent an almost palindromic region within the *ohsC* 3′ region, allowing for *ohsC* in the 5′ to 3′ direction to map to itself in the 3′ to 5′ direction; thus, these examples were excluded. Note that these short *ohsC* matches are found within the single copy of *shoB/ohsC* identified within a specific genome; thus, these genomes possess only one *shoB/ohsC* match.

For the *zor/orz* locus, BLASTn results were used to identify strains with both a *zor* and an *orz* gene, a *zor* gene only, an *orz* gene only, or no detected *zor/orz* genes. This was done by first performing a BLASTn of the *zorO/orzO* locus followed by a BLASTn of either only *zorO* or only *orzO* (see BLASTn query sequences and parameters above). For these queries, results of the searches were compared using a series of Linux command line functions. Briefly, the “grep” function was used to condense all lines of the BLASTn results down to only those containing the symbol “>” which resulted in a list of each assembly, one per line, which matched in part to the query sequence. The two BLASTn results were combined using the “cat” function, alphabetized using the “sort” function, and lines with duplication, indicating the same assembly was matched in both BLASTn searches, were removed using the “uniq -u” function. The remaining assemblies listed after this were those with hits in only one of the BLASTn results. Assemblies that appeared in *zorO/orzO* BLASTn results but not *orzO* BLASTn results were annotated as “*zor* only” and assemblies that appeared in *zorO/orzO* BLASTn results but not in *zorO* BLASTn results were annotated as “*orz* only.” No assemblies were found to appear in *zorO* or *orzO* BLASTn and not *zorO/orzO* BLASTn results. These categorizations along with copy number per assembly were confirmed by manually comparing the initial categorization to the number of alignments and the length of the alignment in the BLASTn results for *zorO/orzO* and *zorP/orzP*. During confirmation of copy number with BLASTn, 15 short hits to ~40 nucleotides of the 3′ end of *orzO* were found. Some of which identified an additional *orz* that lacked a cognate *zor* (“orphan” antitoxins).

### PSI-BLAST and tBLASTn

PSI-BLAST was performed for ZorO on 24 August 2022 on NCBI and tBLASTn on 29 April 2024 (11). tBLASTn was also performed on our custom *E. coli* database using the tBLASTn function. Parameters were set to be as close to those previously found to be optimal for detection of small toxin proteins (21). These included matrix = PAM70, word size = 2, PSI-BLAST inclusion threshold = 1 (for PSI-BLAST only), expect threshold = 100, low complexity filtering turned off, no composition-based statistics. For tBLASTn, the organism of interest was set to *E. coli* only. Gap cost was set to existence 8, extension 2. Following the initial PSI-BLAST, a follow-up PSI-BLAST with the less conserved Zor-translated ORF was performed to search for additional matches. The score cutoff for a match to Zor for tBLASTn was 39.

Translation of tBLASTn results was performed using the Sequence Manipulation Suite Translate tool (38). Translated ORF alignments were performed using Clustal Omega on EMBL-EBI (41), while consensus sequence logos were constructed using WebLogo 3 (40).

### Identification of plasmid localization

To determine if plasmid sequences were identified, the grep function was used on the BLASTn results to identify lines with the word “plasmid” and “extrachromosomal.” Such hits were confirmed by manual observation.

### Analysis of *zor* and *orz* locus for *zorO/orzO, zorP/orzP,* or *zorQ/orzQ* categorization

For categorization of *zor/orz* loci as *zorO/orzO, zorP/orzP*, or *zorQ/orzQ*, a bit score >650 via BLASTn was categorized as the query used because (i) there was no overlap between categorizations using it; (ii) there was a large decrease in bit score between the scores above and below this threshold (decrease for *zorO-orzP* = 163, *zorP/orzP* = 65, and *zorQ/orzQ* = 60); and (iii) all alignments above this cutoff had the highest level of percent identity to the main query over the others. For *orzO* in *orz*-only loci, a bit score cutoff of 280 was used while the cutoff for *orzP* was 250 using the same logic as above.

### Statistical analyses

*t*-Tests were performed for analysis of toxicity and persistence assays using GraphPad Prism version 10.0.3 for Windows, GraphPad Software, Boston, MA, USA, https://www.graphpad.com/.

For analysis of pathotype correlations with copy number, pathotype was determined based on annotation (e.g., EHEC, STEC) or based on isolation source and host disease state (such as human blood or fecal sample and diarrheal disease). If not enough information was provided to categorize by pathotype (such as isolated from human stool but no information if a healthy host), they were excluded from this analysis.

Differences in distribution of gene frequencies among pathotypes for both *zor/orz* and *tisB/istR* pairs were tested via a one-way analysis of variance (ANOVA). Because the sample sizes among pathotypes were imbalanced and variance was not equal among pathotypes, we determined significance by comparing observed *F*-values to the distribution of *F* generated in a Monte Carlo sample of data with randomly shuffled labels, representing the null hypothesis, i.e., no difference in the mean copy number between the pathotypes (42). *Ad hoc* analysis included a Monte Carlo Tukey test to compare means within a pair-wise fashion. We also examined potential copy number distribution per geographical location for *zor-orz*. Our analysis indicated that there are small but significant differences (*P* < 0.03 via ANOVA) between copy number of *zor-orz* from North American isolates (strains were fewer copies) versus those in Asia and Europe details (https://github.com/adsteen/toxin-antitoxin/blob/main/monte-carlo.md, under the heading “zor region diffs”).

A notebook showing R code for all statistical analyses, run using R version 4.3.2, is available at https://githb/adsteen/toxin-antitoxin.

### Bacterial strains

All bacterial strains and plasmids utilized in this work are listed in Table S22. The sequences of all oligonucleotides are listed in Table S23.

*E. coli* UTK007 (derivative of MG1655) Δ*tis/istR* was constructed as described previously via recombineering (43). Briefly, the flippase recognition target (FRT)-flanked kanamycin resistance gene region of plasmid pKD4 (43) was amplified with external homology arms to the region of interest (see Table S22 through 23 for plasmids and primers). The *tis/istR* region in *E. coli* strain NM1100 was replaced with the kanamycin resistance cassette, followed by P1 transduction into *E. coli* UTK007. The kanamycin resistance cassette was then removed via transformation of pCP20 which harbors the FLP recombinase (44), generating *E. coli* Δ*tis/istR*.

*E. coli* MG1655 Δ*orzPmg* was constructed as described previously via recombineering (43). Briefly, the FRT-flanked chloramphenicol resistance gene region of plasmid pKD3 (43) was amplified with external homology arms to the region of interest (see Table S22 to S23 for plasmids and primers). The Δ*orzPmg* gene deletion resulted in the loss of the *orzPmg* promoter and the first 34 nucleotides of *orzPmg* as well as all homology to the region that aligned to the *zorP* promoter and the *zorP* 5′ UTR. The region in *E. coli* strain NM1100 was first deleted, followed by P1 transduction into *E. coli* strain MG1655. The chloramphenicol resistance cassette was removed via transformation of pCP20 which harbors the FLP recombinase (44), generating MG1655 Δ*orzPmg*.

### Plasmid construction

To generate pAZ3-*tisB*-N2S*,* site-directed mutagenesis was performed as described previously on pAZ3-*tisB* using oligonucleotides EF2051 and EF2052 (Table S22) (23, 24, 45). The reaction mixture was digested with DpnI (New England Biolabs), purified using Qiaquick PCR Purification Kit (Qiagen), and then transformed into *E. coli* TOP10 cells (ThermoFisher). Plasmid DNA was extracted via the QiaPrep Spin Miniprep Kit (Qiagen) and confirmed via sequencing.

To generate pBR322-*tis/istRmg*, Gibson assembly was performed as described previously (46). Briefly, the *tis/istR* sequence was amplified from *E. coli* strain MG1655 using oligonucleotides EF2080 and EF2081. Next, pBR322 vector backbone was amplified from pBR322 using oligonucleotides EF2082 and EF2083. These two products were then assembled using the NEB Gibson Assembly Master Mix. The assembled product was transformed into *E. coli* strain TOP10 and sequence verified.

To generate pBR322-*tis/istRmg-tisB*-N2S*,* site-directed mutagenesis was performed as described above on pBR322-*tis/istRmg*, using oligonucleotides EF2051 and EF2052 (Table S22) (23, 24, 45). To generate pAZ3-Δ*28zorO*-CTC*,* site-directed mutagenesis was also performed as described above on pAZ3-Δ*28zorO* (25) using oligonucleotides EF2022 and EF2023 (Table S22) (23, 24, 45).

### Growth conditions

*E. coli* strains were grown at 37°C in lysogeny broth (per liter: 10 g NaCl, 10 g tryptone, 5 g yeast extract) (LB) medium with shaking. Antibiotics were added as warranted at the following concentration: 25 µg/mL chloramphenicol, 30 µg/mL kanamycin, 100 µg/mL ampicillin.

For toxicity assays, strain UTK007 (MG1655 derivative [24]) or UTK007 Δ*tis/istR* was transformed with pAZ3-Δ28*zorO* (25), pAZ3-Δ28*zorO*-CTC, pAZ3-*tisB*, or pAZ3-*tisB*-N2S (23). For pAZ3 plasmids, cultures were grown overnight in LB media supplemented with 0.2% glucose to ensure repression of the P_BAD_ promoter (47). In the morning, cultures were diluted to a final OD_600_ of 0.01. Upon reaching an OD_600_ ~0.25, cultures were divided into two, one serving as a no arabinose control and the other receiving arabinose added to the indicated final concentration. Either OD_600_ values were recorded over time or samples were taken, serial diluted, and plated on LB agar to enumerate colony foming units (CFU) per milliliter. Log ratio of survivors was calculated by taking the ratio of cell counts at each given timepoint to cell counts at timepoint 0 and performing a log transformation. Shown are the averages and standard deviations for *n* = 3.

For persistence assays, a modified persister assay was performed based on those previously described (8). UTK007 Δ*tis/istR* was first transformed with pBR322, pBR322-*tis/istRmg*, or pBR322-*tis/istR-tisB*-N2S. Cultures were grown in Mueller Hinton media supplemented with 10 mg/L MgSO_4_ and 20 mg/L CaCl_2_ overnight and diluted to a final OD_600_ of 0.01 the following morning in the same medium. Upon reaching an OD_600_ ~0.25, cultures were divided into two, one serving as a no ciprofloxacin control and the other receiving ciprofloxacin added to a final concentration of 1 µg/mL. Samples were taken at the indicated timepoints, serially diluted, and plated on LB agar to enumerate CFU per milliliter. Log ratio of survivors was calculated as described above. Shown are the averages and standard deviations for *n* = 3.

### RNA isolation

For detection of OrzPmg sRNA from *E. coli*, overnight cultures of MG1655 derived strains were grown overnight in LB broth and diluted the next morning to an OD_600_ of 0.01. At an OD_600_ of ~0.3 and 24 h post-dilution, cells (15 mL and 10 mL, respectively) were harvested for RNA isolation as previously described (48). RNA integrity was confirmed via gel electrophoresis.

Sample RNA (12 µg) and the Biotinylated sRNA Ladder (Kerafast) were separated on a denatured 8% polyacrylamide-urea gel for detection of OrzPmg and then transferred to a Zeta-Probe Genomic GT Membrane (Bio-Rad). Hybridization (ULTRAhyb Ultrasensitive Hybridization Solution, ThermoFisher) was performed as previously described (49) with the 5′-end labeled biotin-specific oligonucleotide probes listed in Table S22. Detection was based on the previous methodology of the Brightstar Biotect Kit (formerly of Ambion). Briefly, the blots were washed two times in 1× wash buffer (58 mM Na_2_HPO_4_, 17 mM NaH_2_PO_4_, 68 mM NaCl, 0.1% sodium dodecyl sulfate [SDS]), 5 min each. This was followed with three washes (2 for 5 min; final for 1 h) in 1× blocking buffer (58 mM Na_2_HPO_4_, 17 mM NaH_2_PO_4_, 68 mM NaCl, 0.3% casein, 2% SDS) and then incubated with streptavidin alkaline phosphatase conjugate (ThermoFisher; in 1× blocking buffer) for 30–60 min. The blot was then washed once for 10 min with 1× blocking buffer, and three times in 1× wash buffer for 15 min each. This was followed by two 2-min washes in 1× assay buffer (100 mM Tris, 100 mM NaCl, pH 9.5), and then incubation with CDP-STAR (ThermoFisher) for 5 min. Excess reagent was allowed to drip off and the blot was exposed to X-ray film.

## Data Availability

The data underlying this article are available in the article and in its supplemental material.

## SUPPLEMENTAL MATERIAL

Tables S1 to S23 msystems.01204-23-s0001.xlsx Supplemental tables of raw and processed data.

ASM does not own the copyrights to Supplemental Material that may be linked to, or accessed through, an article. The authors have granted ASM a non-exclusive, world-wide license to publish the Supplemental Material files. Please contact the corresponding author directly for reuse.

## References

[1] Singh G, Yadav M, Ghosh C, Rathore JS. 2021. Bacterial toxin-antitoxin modules: classification, functions, and association with persistence. Curr Res Microb Sci 2:100047. doi:10.1016/j.crmicr.2021.100047 34841338 PMC8610362

[2] Masachis S, Darfeuille F. 2018. Type I toxin-antitoxin systems: regulating toxin expression via Shine-Dalgarno sequence sequestration and small RNA binding. Microbiol Spectr 6. doi:10.1128/microbiolspec.RWR-0030-2018 PMC11633621 30051800

[3] Brantl S, Jahn N. 2015. sRNAs in bacterial type I and type III toxin-antitoxin systems. FEMS Microbiol Rev 39:413–427. doi:10.1093/femsre/fuv003 25808661

[4] Vogel J, Argaman L, Wagner EGH, Altuvia S. 2004. The small RNA IstR inhibits synthesis of an SOS-induced toxic peptide. Curr Biol 14:2271–2276. doi:10.1016/j.cub.2004.12.003 15620655

[5] Wen J, Fozo EM. 2014. sRNA antitoxins: more than one way to repress a toxin. Toxins (Basel) 6:2310–2335. doi:10.3390/toxins6082310 25093388 PMC4147584

[6] Sarpong DD, Murphy ER. 2021. RNA regulated toxin-antitoxin systems in pathogenic bacteria. Front Cell Infect Microbiol 11:661026. doi:10.3389/fcimb.2021.661026 34084755 PMC8167048

[7] Brantl S, Müller P. 2019. Toxin-antitoxin systems in Bacillus subtilis. Toxins 11:262. doi:10.3390/toxins11050262 31075979 PMC6562991

[8] Dörr T, Vulić M, Lewis K. 2010. Ciprofloxacin causes persister formation by inducing the TisB toxin in Escherichia coli. PLoS Biol 8:e1000317. doi:10.1371/journal.pbio.1000317 20186264 PMC2826370

[9] Bogati B, Wadsworth N, Barrera F, Fozo EM. 2022. Improved growth of Escherichia coli in aminoglycoside antibiotics by the zor-orz toxin-antitoxin system . J Bacteriol 204:JB0040721. doi:10.1128/JB.00407-21 PMC8765423 34570627

[10] Yasir M, Turner AK, Bastkowski S, Lott M, Holden ER, Telatin A, Page AJ, Webber MA, Charles IG. 2022. Genome-wide analysis of innate susceptibility mechanisms of Escherichia coli to colistin. Antibiotics (Basel) 11:1668. doi:10.3390/antibiotics11111668 36421312 PMC9687012

[11] Sayers EW, Bolton EE, Brister JR, Canese K, Chan J, Comeau DC, Connor R, Funk K, Kelly C, Kim S, Madej T, Marchler-Bauer A, Lanczycki C, Lathrop S, Lu Z, Thibaud-Nissen F, Murphy T, Phan L, Skripchenko Y, Tse T, Wang J, Williams R, Trawick BW, Pruitt KD, Sherry ST. 2022. Database resources of the national center for biotechnology information. Nucleic Acids Res 50:D20–D26. doi:10.1093/nar/gkab1112 34850941 PMC8728269

[12] Darfeuille F, Unoson C, Vogel J, Wagner EGH. 2007. An antisense RNA inhibits translation by competing with standby ribosomes. Mol Cell 26:381–392. doi:10.1016/j.molcel.2007.04.003 17499044

[13] Berghoff BA, Hoekzema M, Aulbach L, Wagner EGH. 2017. Two regulatory RNA elements affect TisB-dependent depolarization and persister formation. Mol Microbiol 103:1020–1033. doi:10.1111/mmi.13607 27997707

[14] Berghoff BA, Wagner EGH. 2019. Persister formation driven by TisB-dependent membrane depolarization, p 77–97. In K L (ed), Persister cells and infectious disease. Springer, Cham.

[15] Weel-Sneve R, Bjørås M, Kristiansen KI. 2008. Overexpression of the LexA-regulated tisAB RNA in E. coli inhibits SOS functions; implications for regulation of the SOS response. Nucleic Acids Res 36:6249–6259. doi:10.1093/nar/gkn633 18832374 PMC2577331

[16] Camacho C, Coulouris G, Avagyan V, Ma N, Papadopoulos J, Bealer K, Madden TL. 2009. BLAST+: architecture and applications. BMC Bioinformatics 10:421. doi:10.1186/1471-2105-10-421 20003500 PMC2803857

[17] Browning DF, Hobman JL, Busby SJW. 2023. Laboratory strains of Escherichia coli K-12: things are seldom what they seem. Microb Genom 9:mgen000922. doi:10.1099/mgen.0.000922 36745549 PMC9997739

[18] Daegelen P, Studier FW, Lenski RE, Cure S, Kim JF. 2009. Tracing ancestors and relatives of Escherichia coli B, and the derivation of B strains REL606 and BL21(DE3). J Mol Biol 394:634–643. doi:10.1016/j.jmb.2009.09.022 19765591

[19] Hobman JL, Penn CW, Pallen MJ. 2007. Laboratory strains of Escherichia coli: model citizens or deceitful delinquents growing old disgracefully? Mol Microbiol 64:881–885. doi:10.1111/j.1365-2958.2007.05710.x 17501914

[20] Kawano M, Reynolds AA, Miranda-Rios J, Storz G. 2005. Detection of 5'- and 3'-UTR-derived small RNAs and cis-encoded antisense RNAs in Escherichia coli. Nucleic Acids Res 33:1040–1050. doi:10.1093/nar/gki256 15718303 PMC549416

[21] Fozo EM, Makarova KS, Shabalina SA, Yutin N, Koonin EV, Storz G. 2010. Abundance of type I toxin-antitoxin systems in bacteria: searches for new candidates and discovery of novel families. Nucleic Acids Res 38:3743–3759. doi:10.1093/nar/gkq054 20156992 PMC2887945

[22] Zhao Z, Xu Y, Jiang B, Qi Q, Tang YJ, Xian M, Wang J, Zhao G. 2022. Systematic identification of CpxRA-regulated genes and their roles in Escherichia coli stress response. mSystems 7:e0041922. doi:10.1128/msystems.00419-22 36069452 PMC9600279

[23] Fozo EM, Kawano M, Fontaine F, Kaya Y, Mendieta KS, Jones KL, Ocampo A, Rudd KE, Storz G. 2008. Repression of small toxic protein synthesis by the Sib and OhsC small RNAs. Mol Microbiol 70:1076–1093. doi:10.1111/j.1365-2958.2008.06394.x 18710431 PMC2597788

[24] Wen J, Won D, Fozo EM. 2014. The ZorO-OrzO type I toxin-antitoxin locus: repression by the OrzO antitoxin. Nucleic Acids Res 42:1930–1946. doi:10.1093/nar/gkt1018 24203704 PMC3919570

[25] Wen J, Harp JR, Fozo EM. 2017. The 5΄ UTR of the type I toxin ZorO can both inhibit and enhance translation. Nucleic Acids Res 45:4006–4020. doi:10.1093/nar/gkw1172 27903909 PMC5397157

[26] Bogati B, Shore SFH, Nipper TD, Stoiculescu O, Fozo EM. 2022. Charged amino acids contribute to ZorO toxicity. Toxins (Basel) 15:32. doi:10.3390/toxins15010032 36668852 PMC9860968

[27] Sterk M, Romilly C, Wagner EGH. 2018. Unstructured 5'-tails act through ribosome standby to override inhibitory structure at ribosome binding sites. Nucleic Acids Res 46:4188–4199. doi:10.1093/nar/gky073 29420821 PMC5934652

[28] Romilly C, Deindl S, Wagner EGH. 2019. The ribosomal protein S1-dependent standby site in tisB mRNA consists of a single-stranded region and a 5' structure element. Proc Natl Acad Sci U S A 116:15901–15906. doi:10.1073/pnas.1904309116 31320593 PMC6690012

[29] Adams PP, Baniulyte G, Esnault C, Chegireddy K, Singh N, Monge M, Dale RK, Storz G, Wade JT. 2021. Regulatory roles of Escherichia coli 5' UTR and ORF-internal RNAs detected by 3' end mapping. Elife 10. doi:10.7554/eLife.62438 PMC7815308 33460557

[30] Thomason MK, Bischler T, Eisenbart SK, Förstner KU, Zhang A, Herbig A, Nieselt K, Sharma CM, Storz G. 2015. Global transcriptional start site mapping using differential RNA sequencing reveals novel antisense RNAs in Escherichia coli. J Bacteriol 197:18–28. doi:10.1128/JB.02096-14 25266388 PMC4288677

[31] Karp PD, Billington R, Caspi R, Fulcher CA, Latendresse M, Kothari A, Keseler IM, Krummenacker M, Midford PE, Ong Q, Ong WK, Paley SM, Subhraveti P. 2019. The BioCyc collection of microbial genomes and metabolic pathways. Brief Bioinform 20:1085–1093. doi:10.1093/bib/bbx085 29447345 PMC6781571

[32] Eisenbart SK, Alzheimer M, Pernitzsch SR, Dietrich S, Stahl S, Sharma CM. 2020. A repeat-associated small RNA controls the major virulence factors of Helicobacter pylori. Molecular Cell 80:210–226. doi:10.1016/j.molcel.2020.09.009 33002424

[33] Sharma CM, Hoffmann S, Darfeuille F, Reignier J, Findeiss S, Sittka A, Chabas S, Reiche K, Hackermüller J, Reinhardt R, Stadler PF, Vogel J. 2010. The primary transcriptome of the major human pathogen Helicobacter pylori. Nature 464:250–255. doi:10.1038/nature08756 20164839

[34] Silvaggi JM, Perkins JB, Losick R. 2005. Small untranslated RNA antitoxin in Bacillus subtilis. J Bacteriol 187:6641–6650. doi:10.1128/JB.187.19.6641-6650.2005 16166525 PMC1251590

[35] Verstraeten N, Knapen WJ, Kint CI, Liebens V, Van den Bergh B, Dewachter L, Michiels JE, Fu Q, David CC, Fierro AC, Marchal K, Beirlant J, Versées W, Hofkens J, Jansen M, Fauvart M, Michiels J. 2015. Obg and membrane depolarization are part of a microbial bet-hedging strategy that leads to antibiotic tolerance. Mol Cell 59:9–21. doi:10.1016/j.molcel.2015.05.011 26051177

[36] Pedersen K, Gerdes K. 1999. Multiple hok genes on the chromosome of Escherichia coli. Mol Microbiol 32:1090–1102. doi:10.1046/j.1365-2958.1999.01431.x 10361310

[37] Le Rhun A, Tourasse NJ, Bonabal S, Iost I, Boissier F, Darfeuille F. 2023. Profiling the intragenic toxicity determinants of toxin-antitoxin systems: revisiting hok/sok regulation. Nucleic Acids Res 51:e4. doi:10.1093/nar/gkac940 36271796 PMC9841398

[38] Stothard P. 2000. The sequence manipulation suite: JavaScript programs for analyzing and formatting protein and DNA sequences. Biotechniques 28:1102, 1104. doi:10.2144/00286ir01 10868275

[39] Waterhouse AM, Procter JB, Martin DMA, Clamp M, Barton GJ. 2009. Jalview version 2--a multiple sequence alignment editor and analysis workbench. Bioinformatics 25:1189–1191. doi:10.1093/bioinformatics/btp033 19151095 PMC2672624

[40] Crooks GE, Hon G, Chandonia JM, Brenner SE. 2004. WebLogo: a sequence logo generator. Genome Res 14:1188–1190. doi:10.1101/gr.849004 15173120 PMC419797

[41] Madeira F, Pearce M, Tivey ARN, Basutkar P, Lee J, Edbali O, Madhusoodanan N, Kolesnikov A, Lopez R. 2022. Search and sequence analysis tools services from EMBL-EBI in 2022. Nucleic Acids Res 50:W276–W279. doi:10.1093/nar/gkac240 35412617 PMC9252731

[42] Edgington E, Edgington E, Onghena P. 2007. Randomization tests. Vol. 4. Chapman & Hall/CRC.

[43] Datsenko KA, Wanner BL. 2000. One-step inactivation of chromosomal genes in Escherichia coli K-12 using PCR products. Proc Natl Acad Sci U S A 97:6640–6645. doi:10.1073/pnas.120163297 10829079 PMC18686

[44] Cherepanov PP, Wackernagel W. 1995. Gene disruption in Escherichia coli: TcR and KmR cassettes with the option of Flp-catalyzed excision of the antibiotic-resistance determinant. Gene 158:9–14. doi:10.1016/0378-1119(95)00193-a 7789817

[45] Ho SN, Hunt HD, Horton RM, Pullen JK, Pease LR. 1989. Site-directed mutagenesis by overlap extension using the polymerase chain reaction. Gene 77:51–59. doi:10.1016/0378-1119(89)90358-2 2744487

[46] Gibson DG, Young L, Chuang R-Y, Venter JC, Hutchison CA III, Smith HO. 2009. Enzymatic assembly of DNA molecules up to several hundred kilobases. Nat Methods 6:343–345. doi:10.1038/nmeth.1318 19363495

[47] Guzman LM, Belin D, Carson MJ, Beckwith J. 1995. Tight regulation, modulation, and high-level expression by vectors containing the arabinose PBAD promoter. J Bacteriol 177:4121–4130. doi:10.1128/jb.177.14.4121-4130.1995 7608087 PMC177145

[48] Kawano M, Oshima T, Kasai H, Mori H. 2002. Molecular characterization of long direct repeat (LDR) sequences expressing a stable mRNA encoding for a 35-amino-acid cell-killing peptide and a cis-encoded small antisense RNA in Escherichia coli. Mol Microbiol 45:333–349. doi:10.1046/j.1365-2958.2002.03042.x 12123448

[49] De Lay N, Gottesman S. 2009. The Crp-activated small noncoding regulatory RNA CyaR (RyeE) links nutritional status to group behavior. J Bacteriol 191:461–476. doi:10.1128/JB.01157-08 18978044 PMC2620814

